# Emotional reactivity and cognitive performance in aversively motivated tasks: a comparison between four rat strains

**DOI:** 10.1186/1744-9081-5-50

**Published:** 2009-12-15

**Authors:** F Josef van der Staay, Teun Schuurman, Cornelis G van Reenen, S Mechiel Korte

**Affiliations:** 1 Programme Emotion & Cognition, Department of Farm Animal Health, Faculty of Veterinary Medicine, University Utrecht, PO Box 80151, 3508 TD Utrecht, the Netherlands; 2 BioMedical Research, Wageningen University and Research Center, Lelystad, the Netherlands; 3 Livestock Research, Wageningen University and Research Center, Lelystad, the Netherlands; 4 Department of Psychopharmacology, Utrecht Institute of Pharmaceutical Sciences (UIPS), Utrecht University, Utrecht, the Netherlands; 5 Rudolf Magnus Institute of Neuroscience, Utrecht University, Utrecht, the Netherlands

## Abstract

**Background:**

Cognitive function might be affected by the subjects' emotional reactivity. We assessed whether behavior in different tests of emotional reactivity is correlated with performance in aversively motivated learning tasks, using four strains of rats generally considered to have a different emotional reactivity.

**Methods:**

The performance of male Brown Norway, Lewis, Fischer 344, and Wistar Kyoto rats in open field (OF), elevated plus-maze (EPM), and circular light-dark preference box (cLDB) tasks, which are believed to provide measures of emotional reactivity, was evaluated. Spatial working and reference memory were assessed in two aversively motivated learning and memory tasks: the standard and the "repeated acquisition" versions of the Morris water maze escape task, respectively. All rats were also tested in a passive avoidance task. At the end of the study, levels of serotonin (5-HT) and *5*-hydroxyindoleacetic acid, and 5-HT turnover in the hippocampus and frontal cortex were determined.

**Results:**

Strain differences showed a complex pattern across behavioral tests and serotonergic measures. Fischer 344 rats had the poorest performance in both versions of the Morris water escape task, whereas Brown Norway rats performed these tasks very well but the passive avoidance task poorly. Neither correlation analysis nor principal component analysis provided convincing support for the notion that OF, EPM, and cLDB tasks measure the *same *underlying trait.

**Conclusions:**

Our findings do not support the hypothesis that the level of emotional reactivity modulates cognitive performance in aversively motivated tasks. Concepts such as "emotional reactivity" and "learning and memory" cannot adequately be tapped with only one behavioral test. Our results emphasize the need for multiple testing.

## Background

Emotion and cognition appear to be closely associated, with emotions affecting learning and memory, and vice versa [1,2]. The influence of emotions, such as fear and anxiety [3,4], on learning and memory is thought to involve the hippocampus and/or the amygdala, structures that modulate both cognitive and emotional processes (e.g. [5,6]). However, experimental evidence does not unambiguously support this hypothesis [3,7,8]. For example, Miyagawa and colleagues [9] reported a dissociation between age-related spatial memory impairments, motor functions, and emotional behavior. Moreover, study of the effect of emotions or experimental manipulations on learning and memory may be confounded by differences in the emotionality of the rats or mice used for such studies [10-12].

### Assessment of emotional reactivity

Several tests are available to assess *unconditioned *emotional reactivity, fear or anxiety, in rodents [2,13], such as the open field, the light-dark preference box, and the elevated plus maze. Unfortunately, none of these tests has been fully standardized, and virtually every laboratory has its own version of the equipment and applies its own experimental procedures and testing protocols.

Although *emotional reactivity *and *anxiety *are not synonymous, because they consist of a number of components that appear to be genetically independent [7], it is not possible to distinguish between the two concepts using the above-mentioned test paradigms and so we use the term "emotional reactivity" throughout this manuscript.

Several variables can be measured in the ***o****pen ****f****ield *(**OF**) as indices of emotional reactivity. For example, a lower number of squares entered [14,15], high defecation scores [15-17], decreased time spent in the center [18], increased occupancy of side squares ('thigmotaxis', [2,19], or more particular of corner squares [20,21], are considered to indicate higher emotional reactivity.

The ***e****levated ****p****lus ****m****aze *(**EPM**) has been extensively validated as test of emotional reactivity in rodents [22,23]. An increased occupancy of the closed arms and a decreased occupancy of the open arms are considered to indicate higher emotional reactivity, whereas the number of closed arm entries is considered to reflect activity that is independent of emotional reactivity [24]. The EPM varies greatly between laboratories, with technical modifications, such as the presence or absence of ledges around the perimeter of the open arms [25], or procedural modifications such as introduction of additional stressors [26,27], handling or testing in another apparatus such as the holeboard [28] prior to testing [29], that may affect the results obtained with the EPM.

The ***c****ircular ****l****ight-****d****ark preference ****B****ox *(**cLDB**) is our variant of the *light-dark preference box *[14,21,30,31] designed to assess the effects of experimental manipulations on emotional reactivity in rats. In general, light-dark preference tests are based on the observation that, given the choice, a rat 'prefers' to stay in the unlit rather than lit part of a test environment. Rats that stay in the dark for a long time and that show little ambulatory activity are considered emotionally reactive [14]. The cLDB lacks spatial cues for the light compartment, the transition between light and dark, and the dark compartment [12].

### Assessment of learning and memory in aversively motivated tasks

Aversively motivated tasks trigger emotional reactivity, eliciting an escape or avoidance response [32]. We used three aversely motivated tasks, two of them variants of the Morris water escape task. The Morris tasks provide reinforcement by allowing the rat to escape from the aversive water onto a submerged platform [33]. The ***s****tandard ****M****orris ****w****ater ****m****aze (****sMWM****) escape task *[34,35] can be used to measure spatial reference memory in rats and mice. Reference memory holds trial-independent information [36] about the position of the escape platform in the water tank. Variants of the Morris water escape task have been developed that allow assessment of a working memory or short-term memory component of spatial memory [37-42]. Whishaw [43,44] described a repeated acquisition paradigm to test the formation of what he called a *place learning set *by rats. Within a daily training session of this ***r****epeated ****a****cquisition ****M****orris ****w****ater ****m****aze *(**raMWM**)* escape task*, each of four start positions (situated in the northern, eastern, southern, or western quadrant of the pool) is used randomly in each series of four trial pairs.

We used an inhibitory or ***p****assive ****a****voidance *(***PA***)* task *to measure 24-hour retention of an aversive event. In this task, a rat learns to avoid the dark compartment that had gained aversive properties because the rat had received a mild electric footshock in that compartment 24 hours earlier. The latency to enter the dark compartment during the retention session in the PA task is usually interpreted as a measure of *conditioned *anxiety [45].

A number of studies have shown that drugs that reduce serotonin (5-hydroxytryptamine; 5-HT) function produce anxiolytic-like effects. 5-HT 1A receptor ligands reduce serotonergic function by acting as agonists at somatodendritic autoreceptors, which inhibit the firing of 5-HT neurons in the raphe nuclei [46-49]. 5-HT turnover (5-HIAA/5-HT ratio) is decreased, and decreased 5-HT turnover in the dorsal hippocampus has been shown to be associated with an anxiolytic-like effect in the EPM test [50].

### Rat strains

We used four strains of rats, namely, Brown Norway (BN), Lewis, Fischer 344 (F344), and Wistar Kyoto (WKY), to investigate whether there are strain differences in emotional reactivity, aversively motivated learning and memory functions, and serotonergic measures in the hippocampus and cortex. The WKY [51,52], Lewis [53], and F344 [54] strains have been proposed as animal models of anxiety and depression, and are the most-used strains in studies of anxiety [45]. The WKY rat in particular shows exaggerated behavioral and endocrine responses to stressful events [52,55]. Ramos and colleagues [56] suggested that Lewis rats could serve as a genetic animal model of anxiety because of their strong avoidance of the white compartment of a black and white box, and the low proportion of time spent in the open arms of an EPM. In contrast, BN rats show a low responsiveness to stressful stimulation, both physiologically and behaviorally [57-60], and these rats perform poorly in shock-motivated passive [12] and active avoidance tasks [61], but perform well in spatial discrimination tasks such as the Morris task [62] and the hole board task [63]. The F344 and BN strains (and their F1s) have been recommended as standard strains for aging research [64,65].

### Aims of the study

The first aim was to corroborate the construct validity of the OF, EPM, and cLDB tests. To this end, we used the four rat strains that were expected to differ in emotional reactivity [45] and correlated measures between tests. The second aim was to determine whether these rat strains had a different learning and memory performance in the aversively motivated Morris water escape tasks and in the passive avoidance task. Lastly, we evaluated whether performance on the different tests of emotional reactivity was correlated with performance on aversively motivated learning tasks, to determine whether cognition is affected by emotional processes.

## Methods

The study was reviewed and approved by the local ethics committee (DEC, **d**ier**e**xperimenten **c**ommissie), and was conducted in accordance with the recommendations of the EU directive 86/609/EEC. All effort was taken to minimize the number of animals used and their suffering.

### Animals

**Figure 1:**
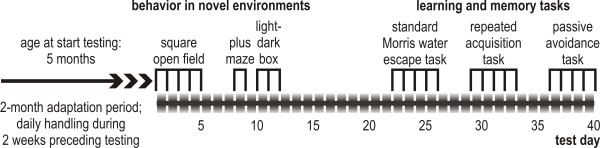
**Time line of the study**. The rats were purchased at the age of approximately 3 months and allowed to adapt to our laboratory for 2 months before behavioral testing started.

Male Brown Norway (BN/Crl), Lewis (LEW/Crl), Fischer 344 (F344/NCrl), and Wistar Kyoto (WKY/NCrl) rats were purchased from Charles River (Sulzfeld, Germany) at the age of approximately 3 months. None of the selected strains suffers from gross locomotor and/or sensory deficits. The rats were allowed to habituate to the animal facilities for 2 months before behavioral testing started at the age of about 5 months. The rats were housed in pairs in Makrolon™ type IV cages in a temperature- (approx. 20°C) and humidity- (approx 60%) controlled laboratory, with food and water available *ad libitum*. Lights were on from 7:00 to 19:00. The time line of the study is depicted in Fig. 1. All testing was performed in the room where the animals were housed. No other animals than the ones used in this study were present in the room.

### Open-field

#### Apparatus

**Figure 2:**
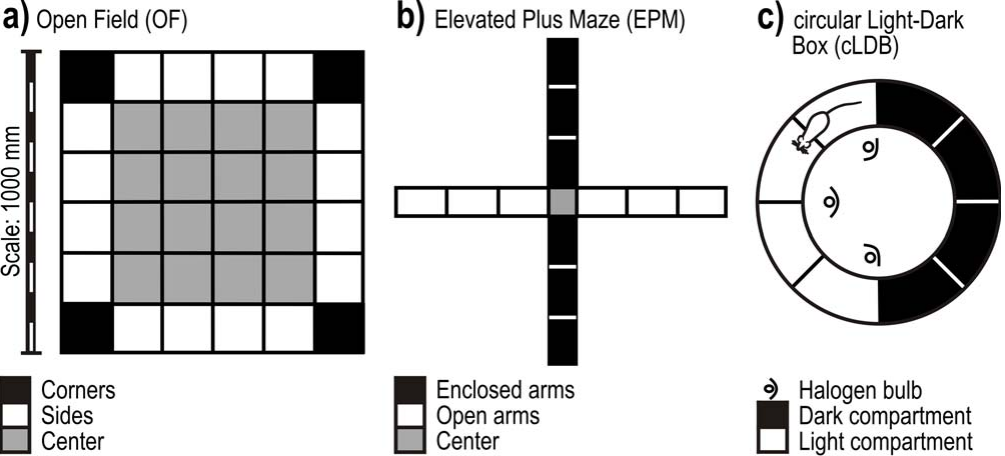
**Maps of the Open field, Elevated Plus Maze and circular Light-Dark Box**. The different parts of the apparatus are shown for the OF (panel a), the EPM (panel b), and the cLDB (panel c).

OF behavior was assessed in a square base (1000 × 1000 mm, height of side walls 400 mm) subdivided into 36 equal squares by black lines (see Fig. 2a). The base and three of the side walls of the OF were made of gray polyvinylchloride (PVC); the last side wall was made of transparent PVC. Testing was carried out under dim illumination (about 5 lux on the floor of the apparatus).

#### Procedures

Immediately after a rat was placed in the center of the OF, its movements were scored. The number of segment entries and the number of line crossings in the different segments of the apparatus (corners, sides, center) were recorded manually, as were the number of bouts of rearing and leaning, and grooming, using the program OBSERVE which runs on an MS-DOS compatible personal computer. The rats were tested on 5 consecutive days in 5-minute sessions.

Five measures of the OF test were analyzed statistically: time spent in the corner squares (in s), total distance moved (expressed as number of line crossings, [66,67]), number of grooming bouts, number of rearings and leanings, and number of fecal boli eliminated [12].

### Elevated plus-maze

#### Apparatus

The EPM was made of gray PVC and consisted of two open arms (length 500 mm, width 100 mm) and two enclosed arms (length 500 mm, width 100 mm, height of side walls 400 mm); the EPM had an open roof [23] (see Fig. 2b). There was a center square in the middle of the maze, and each arm was divided into three equal segments. Two dimmed spotlights provided illumination on the floor of the enclosed and open arms of approximately 5 lux. The apparatus was provided with a 15-mm high ledge along the perimeter of the open arms. The plus maze was elevated 500 mm above the floor.

#### Procedures

The rat was placed on the center square at the start of the trial, and its movements in the apparatus were scored according to File [24], with the same software as used in the OF test. The rats were tested on two consecutive days in 5-minute sessions, i.e. applying a test-retest protocol [68]. In the EPM test, the measures analyzed were percent time spent in open arms, percent open arm entries [23], number of grooming bouts, number of rearings and leanings, and number of fecal boli eliminated.

### Circular light-dark preference box

#### Apparatus

The cLDB consisted of a circular alley (see Fig. 2c). The inner wall (diameter 430 mm) and outer wall (diameter 830 mm) were 350 mm high. Half of the inner wall and half of the outer wall were made of transparent PVC and the other halves were made of black PVC. Three dimmable 50-watt halogen bulbs positioned outside the inner circle of the transparent inner wall provided a light intensity of 800 lux in the light compartment (the section made of transparent PVC) and a light intensity of about 5 lux in the dark compartment (the section made of black PVC). Both the light and the dark compartments were subdivided into four equally spaced segments that were marked with lines on the floor. During testing, the experimental room was illuminated dimly by fluorescent tubes and the light that emanated from the light compartment.

#### Procedures

An observation session started when a rat was placed at the junction between the light and dark compartment: half of the rats were randomly assigned to face the dark compartment and the other half to face the light compartment. A line crossing was scored whenever a rat stepped over the line with both hind legs, and a compartment entry was scored when a rat stepped into it with both hind legs. The number of compartment entries and the number of line crossings in the various segments of the apparatus were recorded manually by the experimenter, using the program OBSERVE. The experimenter sat in front of the cLDB and registered the rat's behavior via a mirror, which was mounted on a holding device. The apparatus was cleaned with a damp sponge after each test. The rats were tested in 5-minute sessions on three consecutive days.

Five measures were analyzed statistically: time spent in the dark compartment, total distance moved (expressed as number of line crossings), number of grooming bouts, number of rearings and leanings, and number of fecal boli eliminated [12].

### Standard Morris water escape task assessing spatial reference memory

Training in sMWM escape task was started when the rats were approximately 6 months old.

#### Apparatus

The Morris water tank and the escape platforms were made of gray polypropylene (PP). The color of the maze and escape platform very closely resembles the gray defined by RAL 7032. The dimensions of the tank were diameter 1700 mm, depth of tank 450 mm, water level 260 mm, diameter of platform 110 mm, and height of platform 250 mm. The center of the platform and of the four annuli were located half the radius of the maze from the rim of the tank.

The water in the Morris tank was not made opaque because the gray escape platform in the gray tank is virtually invisible to swimming rats. During testing, the room was indirectly illuminated by desk lamps directed against the walls. Abundant extra-maze cues were provided by the furniture in the room, including desks, computer equipment, and the presence of the experimenter. All testing was done between 8:00 and 15:00. The movements of the rat were registered automatically by a video-tracking system (EthoVision^® ^for Windows, v2.3, Noldus Information Technology, Wageningen, The Netherlands; [69]) and stored in a personal computer.

#### Procedures

##### Standard acquisition

The animals received four trials during five daily acquisition sessions. The trial started when a rat was placed in the pool, facing the wall of the tank. Each starting position was used once, in random order. The escape platform was always in the same quadrant (north). A trial was terminated as soon as the rat had climbed onto the escape platform or when 90 seconds had elapsed, whichever event occurred first. A rat was allowed to stay on the platform for 30 seconds. Then it was taken from the platform and the next trial was started. If a rat did not find the platform within 90 seconds it was put on the platform by the experimenter and was allowed to stay there for 30 seconds. After completion of the fourth trial (except on the fifth day after completion of the probe trial, see below), the rat was gently dried with crêpe paper and returned to its home cage. The animal was kept warm under a 150-Watt infrared bulb, fixed about 400 mm above the cage.

##### Probe trial

After the fourth trial of the fifth session, a probe trial was given. The platform was removed and the time the rat spent in the four quadrants and annuli was measured for 60 seconds. In the probe trial, all rats started from the same start position, opposite to the quadrant where the escape platform had been positioned during acquisition. Four different measures were taken to evaluate the performance of the rats during acquisition training: escape latency, distance traveled, distance to platform, and swimming speed.

• Escape latency is the time (s) taken to find and escape onto the submerged platform [34].

• Distance traveled (cm) is the total distance swum to find and escape onto the submerged platform [34].

• Distance to platform (cm) [70] is calculated as the mean distance to the platform across all samples drawn by the video-tracking system between the start of a trial and the moment the rat climbs onto the platform.

• Swimming speed (cm.s^-1^) is calculated as distance traveled (cm) divided by escape latency (s). Alternatively, we also calculated swimming velocity as the average distance (> 5 cm) swum in 1 second ([69] see also *Noldus News*, 1996/2). This measure is perhaps more appropriate if the rat spends long periods immobile or floating.

##### Probe trial

For the probe trial, time (s) in quadrants, distance (cm) traveled in quadrants, time in annuli (s), distance (cm) traveled in annuli, time (s) at platform positions, and distance (cm) traveled at platform positions [71,72] were analyzed.

### Repeated acquisition Morris water escape task assessing spatial working memory

After acquisition of the sMWM escape task, the animals were trained on the raMWM task.

#### Procedure

The animals were trained on five successive days, receiving four trial pairs a day (i.e. 8 trials; see Fig. 1 in [41]). Within a daily training session, each of the four start positions was used randomly in each series of four trial pairs. Thus, a rat was randomly started from each of the four starting positions per testing session. From one daily session to the next, the escape platform was moved to another quadrant in the following order: east, south, west, north, and east. When all rats had completed a first trial pair, a second pair was given, etc., until all rats had received four trial pairs. The trial pairs were separated by an interval of approximately 60-90 minutes within each testing session. A trial was terminated as described for the sMWM task. After completion of the second trial of a pair, the rat was dried with crêpe paper and was returned to its home cage until the next trial pair was given.

For each rat, the escape latency, distance traveled, distance to platform, and swimming speed were averaged per session separately for the first and second trials of the pairs.

The average of first swims, i.e. the odd trials, was calculated as:

The average of second swims, i.e. the even trials, was calculated as:

The first subscript represents the number of the trial pair within a session; the second subscript represents the trial within trial pairs.

### The passive or inhibitory avoidance task to assess long-term memory

After completion of the Morris water escape tasks, all rats were tested in the PA task.

#### Apparatus

The apparatus consisted of a two-compartment box with a light compartment and a dark compartment, each measuring 270 (depth) × 370 (width) × 360 (height) mm. The apparatus was made of black plastic, except for the sidewalls of the light compartment, which were white. The floor consisted of a metal grid (diameter of stainless steel bars 6.3 mm; free space between bars 11.3 mm) connected to a shock scrambler. A guillotine door that could be raised 90 mm separated the two compartments. A threshold of 20 mm marked the border between the two compartments when the guillotine door was raised. When the door was open, the illumination in the dark compartment was about 2 lux. The light intensity was about 500 lux at the center of the floor of the light compartment.

#### Procedure

Two habituation sessions, one shock session, and a retention session were given, separated by intersession intervals of 24 hours. In the habituation sessions and the retention session, the rat was allowed to explore the apparatus for 300 seconds. The rat was placed in the light compartment, facing the wall opposite to the guillotine door. After an accommodation period of 15 seconds, the guillotine door was opened so that all parts of the apparatus could be visited freely. In the shock session the guillotine door between the compartments was lowered as soon as the rat had entered the dark compartment with its four paws, and a scrambled 1 mA footshock was administered for 2 seconds. The rat was removed from the apparatus 10 seconds after shock termination and put back into its home cage. The procedure during the retention session was identical to that of the habituation sessions.

The step-through latency, namely, the latency to first enter the dark compartment (in seconds), was analyzed statistically. If the rat did not enter the dark compartment, the step-through latency was ascribed the value 300 seconds. As the latency had a heavily skewed distribution, it was logarithmically transformed [log_10 _(s+1)] before statistical analysis.

### Serotonin in hippocampus and cerebral cortex

The brains of the rats were removed within 1 minute of decapitation and immediately frozen in a dry ice precooled tube containing *n*-heptane and stored at -70°C until the assays were performed. Punches were taken from the hippocampus and frontal cerebral cortex. These samples were used for the measurement of serotonin (5-hydroxytryptamine; 5-HT) and the 5-HT metabolite 5-hydroxyindoleacetic acid (5-HIAA). 5-HT turnover was expressed by the 5-HIAA.5-HT^-1 ^ratio [73].

All determinations were performed by *Brains on-Line *(Groningen, The Netherlands). Frozen tissue samples were weighed in 2-mL Potter tubes. HClO_4 _(0.1 M 0.5 mL) was added and the tissue was homogenized in a motor-driven glass-Teflon Potter homogenizer (Janke & Kinkel KG) at 500 rpm. Homogenates were centrifuged at 14,000 rpm at 4°C for 10 minutes. The resulting supernatant was pipetted off and 20 μL was injected into a reverse-phase/ion pair high-performance liquid chromatography (HPLC) system with electrochemical detection for the measurement of 5-HT and 5-HIAA. The HPLC system consisted of a Shimadzu LC-10Advp HPLC-pump, a Gilson 234 autoinjector, an Antec Decade potentiostat (Antec, Leiden, The Netherlands) with its glassy carbon cell set at +500 mV vs. Ag/AgCl and a Supelco LC-18-DB column (150 mm × 4.6 mm i.d., 3 μm particle size). The mobile phase consisted of an acetic acid/acetate buffer pH 4.1 containing 100 mg.L^-1 ^EDTA, 140 mg.L^-1 ^sodium octyl sulphonate, and 75 mL.L^-1 ^methanol. A flow rate of 1.0 mL.min^-1 ^was used.

### Statistical analyses

#### Behavior in the OF, EPM, and cLDB

Strain differences in these three tests were evaluated statistically by analysis of variance (ANOVA) with the factor Strains and the repeated measures factor Sessions (SAS GLM-procedure; [74,75]), supplemented with one-way ANOVAs of the orthogonal trend coefficients calculated over the successive sessions, and with one-way ANOVAs per session. Orthogonal trend coefficients are tools to describe the learning curves and to assess whether the shapes of these curves are different between strains.

#### Acquisition of the sMWM task

The measures were averaged per rat within each session. Strain differences in the acquisition of the water escape task were assessed by ANOVA with the factor Strains and the repeated measures factor Sessions (sessions 1 to 5 of training), supplemented with one-way ANOVAs of orthogonal trend coefficients calculated over the successive sessions. In addition, strain differences per sessions were analyzed by one-way ANOVA.

#### Probe trial in the sMWM task

Strain differences were assessed with a repeated measures ANOVA with the factor Strains and the repeated measures factor Quadrants, Annuli, or Platform positions (north, east, south, and west are considered as levels of the repeated measures factor), complemented by ANOVAs on the swimming times and distances traveled per quadrant, annulus or platform position.

#### Acquisition of the raMWM task

Strain differences were analyzed by an ANOVA with the factor Strains, and the repeated measures factors Sessions (sessions 1 to 5 of training), and Trial Pairs (average of odd vs. average of even trials within a session, calculated as described above). In addition, strain differences were analyzed by one-way ANOVA per session.

#### Retention in the PA task

Strain differences were assessed by one-way ANOVA.

#### Serotonergic measures in the hippocampus and cerebral cortex

Strain differences in cortical and hippocampal 5-HT and 5-HIAA levels and in 5-HT turnover were assessed by one-way ANOVA.

Supplementing all one-way ANOVAs, we performed, Sidak post-hoc comparisons where appropriate to analyze the differences between strains in more detail.

#### Correlation analysis

We selected 17 variables and derived measures for which there were strain differences and used them in a Spearman and Pearson correlation analysis. The variables were OF time in corners (mean of 5 days), OF line crossings (mean of 5 days), OF rearings (mean of 5 days), EPM percent time in open arms (day 1), EPM percent entries open arms (day 1), EPM rearings (day 1), cLDB time in the dark (mean of 3 days), cLBD line crossings (mean of 3 days), cLDB rearings (mean 3 days), sMWM distance swum (mean of 5 days), sMVM distance swum (linear trend over 5 days), sMWM probe trial time in training annulus, raMVM delta (odd-even trials; mean of 5 days), PA retention (log latency to enter the dark compartment), 5-HT turnover in hippocampus, and 5-HT and 5-HIAA levels in the cortex.

#### Principal component analysis (PCA)

PCA was used to examine patterns of intercorrelations [76] between the 17 variables. Principal components produced by PCA are linear combinations of the original measures reflecting independent characteristics (or dimensions) underlying the correlation matrix. The first component explains most of the variance (expressed in terms of the first eigenvalue), the second component explains most of the remaining variation, and so forth. The loading of each measure on a principal component represents the correlation between the latent characteristic and the original measure and thus indicates the importance of a measure for a principal component. Measures with high loadings on the same principal component of the same sign are positively correlated, and loadings of the opposite sign are negatively correlated. In the context of the assessment of emotional reactivity of animals, principal components obtained with PCA are thought to reflect underlying traits such as emotional reactivity or locomotor activity [25,77-79].

After extraction, principal components were scaled by their standard deviations (square roots of associated eigenvalues) and subjected to varimax rotation. Factors with eigenvalues larger than 1 were retained for further consideration. In addition to a PCA on the observed data, we also performed the same PCA on residuals of an analysis of variance model with strain as a fixed effect. This latter PCA allowed us to examine the correlation structure adjusted for strain. Residuals of fractions (i.e., EPM percent time spent in open arms, EPM percent entries open arms) were obtained using a logistic regression model, comprising a multiplicative overdispersion factor with respect to the binomial variance function. To calculate the residuals of non-normally distributed count data (i.e., EPM rearing on day 1), we used a log linear model comprising a multiplicative dispersion factor relative to the Poisson variance function [80].

## Results

### Open field

#### Time spent in the corners of the OF (see Fig. 3a)

**Figure 3:**
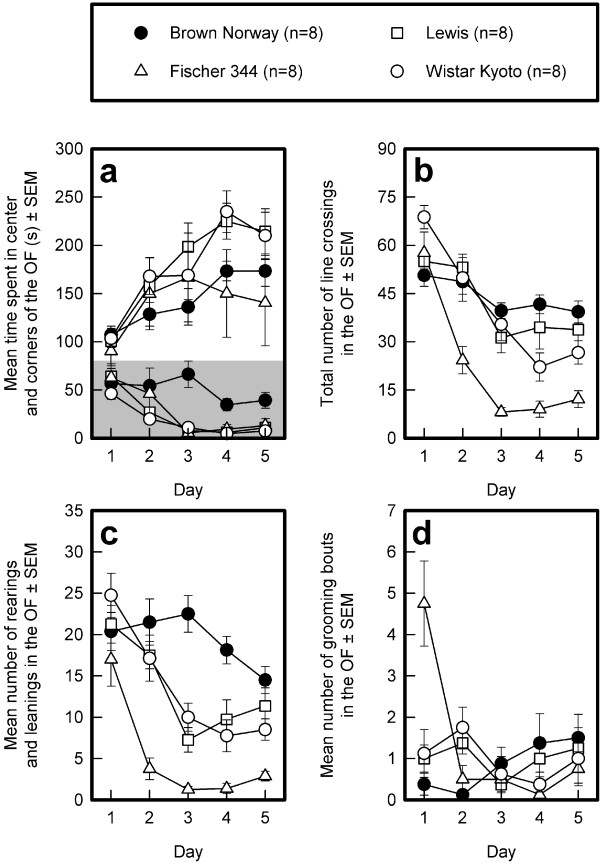
**Open field behavior of four different rat strains**. The means and standard errors of the means (SEMs) of the BN, Lewis, F344, and WKY strains across 5 successive days of testing are depicted for time spent in the corners of the OF (panel a), total number of line crossings (panel b), number of rearings and leanings (panel c), and number of grooming bouts (panel d). Note: the mean time spent in the center of the open field is also depicted in panel a (highlighted grey).

Averaged over the five successive daily sessions, the time spent in the corners tended to be different in the four strains (General mean: F_3,28 _= 2.34, 0.10 > p > 0.05). All strains increased the time spent in the corners across sessions (Sessions: F_4,112 _= 10.16, p < 0.0001) to a similar degree (Sessions by Strains interaction: F_12,112 _= 0.73, NS).

#### Number of line crossings (see Fig. 3b)

The mean number of line crossings, an index for the distance traveled in the OF, was different in the four strains (General mean: F_3,28 _= 21.54, p < 0.0001). Post-hoc Sidak comparisons revealed that, on average, the F344 rats traveled a shorter distance than the other three strains, which did not differ from one another. The distance traveled decreased across successive sessions (Sessions: F_4,112 _= 42.27, p < 0.0001) and to a different extent in the different strains (Sessions by Strains interaction: F_12,112 _= 3.94, p < 0.0001). About 80% of the decrease in the distance traveled could be explained by the linear trend component, which differed by strain (F_3,28 _= 8.19, p < 0.0005). Post-hoc comparisons of the linear decrease confirmed that the distance traveled decreased only slightly in the BN rats and Lewis but much more in the F344 and the WKY rats. The latter two strains did not differ from one another or from the Lewis rats, whereas they differed from the BN. rats.

#### Rearings and leanings (see Fig. 3c)

The average number of rearings and leanings differed in the different strains over the successive sessions (General mean: F_3,28 _= 22.43, p < 0.0001). Post-hoc comparisons revealed that, on average, the BN rats reared the most and the F344 rats the least. Rearing behavior changed across the 5 days of testing (Sessions: F_4,112 _= 32.14, p < 0.0001) to a different extent in the four strains (Sessions by Strains interaction: F_12,112 _= 4.23, p < 0.0001). About 82% of the variation in the change across sessions was explained by the linear trend component, which differed by strain (F_3,28 _= 2.94, p ≤ 0.05). Post-hoc Sidak comparisons of this trend component revealed that the decrease in rearing across sessions was smallest in the BN rats and greatest in the WKY rats. The decrease in rearing across sessions was intermediate in the Lewis and F344 rats and did not differ from that of the BN and the WKY rats.

#### Grooming bouts (see Fig. 3d)

The number of grooming bouts did not differ in the rat strains (General mean: F_3,28 _= 0.47, NS) but did change across the five successive sessions (Sessions: F_4,112 _= 5.39, p < 0.0005), differently for the different strains (Sessions by Strains interaction: F_12,112 _= 7.03, p < 0.0001). Approximately 72% of the change across sessions was explained by the quadratic trend component, which differed by strain (F_3,28 _= 9.59, p < 0.0005). Post-hoc comparisons revealed that the quadratic trend component of the F344 strain was larger than that of the other three strains, which did not differ from one another. In particular, the F344 rats groomed about four times more often than did the other rat strains during the first session (F_3,28 _= 10.01, p < 0.0001). This difference was smaller in the second session (F_3,28 _= 5.24, p < 0.01) and was no longer present in sessions 3 (F_3,28 _= 0.31, NS), 4 (F_3,28 _= 1.64, NS), and 5 (F_3,28 _= 0.38, NS).

#### Defecation

Only 1 of the 8 rats of the Lewis and WKY strains defecated in the OF.

### Elevated plus maze

#### Percent time in open arms of the EPM (see Fig. 4a)

**Figure 4:**
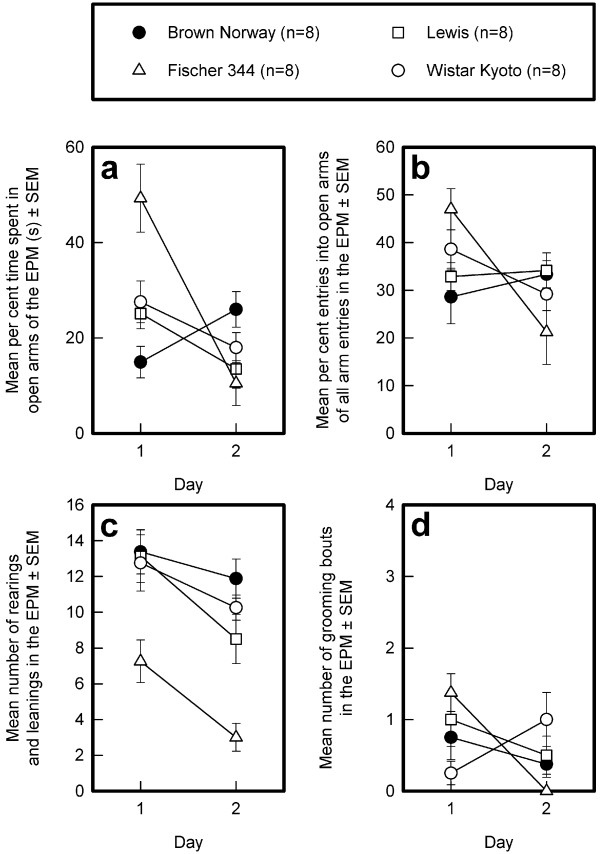
**Strain differences in the elevated plus maze**. The means and standard errors of the means (SEMs) of the BN, Lewis, F344, and WKY strains across 2 successive days of testing are depicted for the percent time spent on the open arms (panel a), percent entries into open arms of all arm entries (open and enclosed arms) (panel b), number of rearings and leanings (panel c), and number of grooming bouts (panel d).

In the first session, the percent time spent in the open arms was different in the four strains (F_3,28 _= 9.15, p < 0.0002). Post-hoc Sidak comparisons revealed that the F344 rats spent more time in the open arms than the other three strains, which did not differ from one another. The strain difference was no longer present on the second day of testing (F_3,28 _= 2.92, NS). Repeated measures ANOVA revealed that, averaged over the two sessions, the percent time spent in open arms did not differ (General Mean: F_3,28 _= 1.63, NS). However, there was an effect of session (Sessions: F_1,28 _= 27.93, p < 0.0001; Sessions by Strains interaction: F_3,28 _= 19.53, p < 0.0001), indicating that the change in this variable from the first to the second session was different in the different rat strains. Post-hoc Sidak comparisons of the difference scores between session 1 and 2 revealed that the time spent in the open arms decreased more in the F344 rats than in the Lewis and WKY rats, which did not differ from one another. The BN rats differed from all other strains. Visual inspection of Fig. 4a suggests that the BN rats spent more time in the open arms in the second session but this was not confirmed by one-sample *t*-statistics on the difference scores (t_7 _= -1.74, NS).

#### Percent open arm entries (see Fig. 4b)

The percent open arm entries was different in the four strains in the first (F_3,28 _= 3.39, p < 0.05), but not the second, session (F_3,28 _= 1.71, NS). Post-hoc analysis of the data of the first session confirmed that the F344 rats made more open arm entries than did the Lewis and BN rats, but not the WKY rats. The latter did not differ from the Lewis and BN rats. Repeated measures ANOVA revealed that the percent open arm entries, averaged over the two sessions, did not differ (General Mean: F_3,28 _= 0.18, NS). There was, however, an effect of session (Sessions: F_1,28 _= 6.99, p < 0.0133), indicating that the change in this variable from the first to the second session was different in the four strains (Sessions by Strains interaction: F_3,28 _= 6.17, p < 0.0024). Post-hoc Sidak comparisons of the difference scores between session 1 and 2 revealed that the percent open arm entries decreased similarly in the F344 and WKY rats (they did not differ from one another) and differently in the Lewis and BN rats (which did not differ from none another). One-sample *t*-statistics on the difference scores confirmed the decrease for the F344 (t_7 _= 3.74, p < 0.0072) and WKY (t_7 _= 3.13, p < 0.0166) rats, whereas there was no change for the percent open arm entries between session 1 and 2 in the BN (t_7 _= -0.67, NS) and Lewis (t_7 _= -0.32, NS) rats.

#### Rearings and leanings in the EPM (see Fig. 4c)

The number of rearings differed by strain on the first (F_3,28 _= 4.51, p < 0.05) and second (F_3,28 _= 14.23, p < 0.0001) day of testing. Post-hoc comparisons confirmed that, on the first day, the F344 rats reared less frequently than the BN and Lewis rats, but not the WKY rats. On the second day, the F344 rats reared less than the other three strains, which did not differ from one another. Repeated measures ANOVA confirmed that, averaged over the two sessions, the number of rearings and leanings was different between strains (General Mean: F_3,28 _= 14.72, p < 0.001). The number of rearings and leanings slightly decreased from the first to the second session (F_1,28 _= 14.52, p < 0.0001), but to a similar extent, in all four strains (Sessions by Strains interaction: F_3,28 _= 0.76, NS).

#### Grooming bouts (see Fig. 4d)

The number of grooming bouts was very low in all four strains, and statistically reliable strain differences were not observed in either the first (F_3,28 _= 2.64, NS) or second (F_3,28 _= 2.75, NS) session.

#### Defecation

None of the rats defecated on the first day of testing in the EPM. On the second day, 3 BN and 2 F344 rats defecated.

### Circular light-dark box

#### Time spent in the dark compartment of the cLDB (see Fig. 5a)

**Figure 5:**
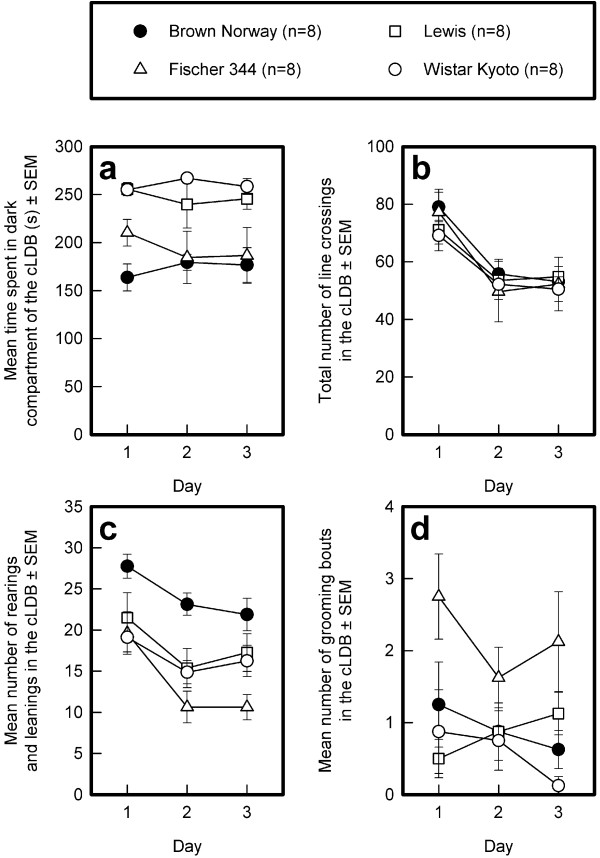
**Strain differences in the circular light-dark box**. The means and standard errors of the means (SEMs) of the BN, Lewis, F344, and WKY strains across 3 successive days of testing are depicted for time spent in the dark compartment (panel a), total number of line crossings (panel b), number of rearings and leanings (panel c), and number of grooming bouts (panel d).

The average time spent in the dark compartment was different in the four strains (General mean: F_3,28 _= 13.27, p < 0.0001). Post-hoc Sidak comparisons revealed that the WKY and Lewis rats spent more time in the dark compartment than did the F344 and BN rats. This strain difference was stable across the 3 days of testing (Sessions: F_4,112 _= 0.10, NS; Sessions by Strains interaction: F_12,112 _= 0.56, NS).

#### Number of line crossings (see Fig. 5b)

The strains did not differ in the number of line crossings, averaged over the three successive sessions (General mean: F_3,28 _= 0.21, NS). The number of lines crossed decreased over sessions (Sessions: F_2,56 _= 27.75, p < 0.0001) in a similar fashions in all strains (Sessions by Strains interaction: F_6,56 _= 0.40,. NS).

#### Rearings and leanings (see Fig. 5c)

The mean number of rearings and leanings was different in the four strains (General mean: F_3,28 _= 7.08, p < 0.005). Post-hoc comparisons confirmed that the BN rats reared more than the WKY and F344 rats, but not more than the Lewis rats. The latter three strains did not differ from one another. Rearings decreased over the three successive sessions (Sessions: F_2,56 _= 22.64, p < 0.0001) to a similar extent in the four strains (Sessions by Strains interaction: F_6,56 _= 1.16, NS).

#### Grooming bouts (see Fig. 5d)

The strains differed in the mean number of grooming bouts across days of testing (General mean: F_3,28 _= 6.80, p < 0.005). The F344 rats groomed more than the other three strains, which did not differ from one another, as confirmed by Sidak post-hoc comparisons. The number of grooming bouts did not change across sessions (Sessions: F_2,56 _= 0.75, NS, Sessions by Strains interaction: F_6,56 _= 0.93, NS)

#### Defecation

Three BN rats and 1 F344 rat defecated on the first day of the testing in the cLDB, and 3 BN rats defecated on the second day of testing. None of the rats defecated on day 3.

### Standard Morris water escape task

#### Acquisition, platform escape latency (see Fig. 6a)

**Figure 6:**
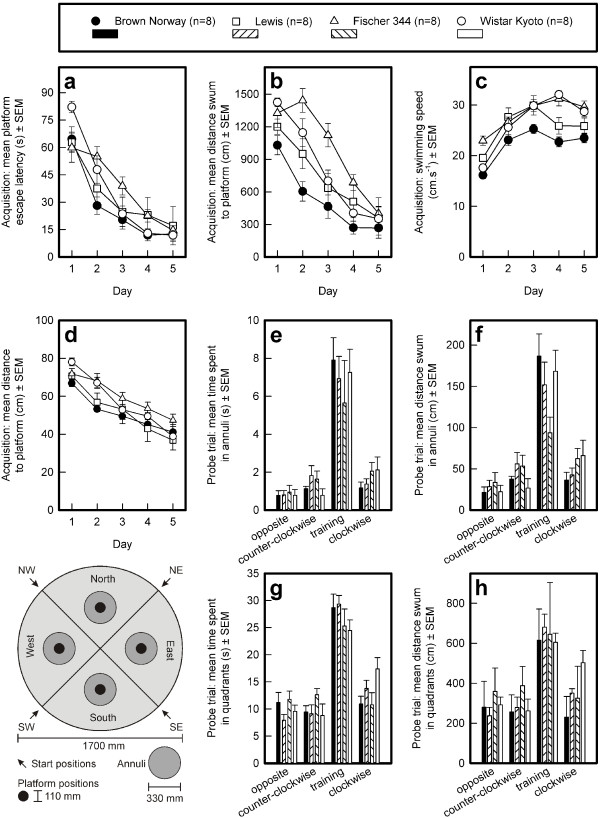
**Acquisition of the standard Morris water escape task by four rat strains**. The platform escape latencies (s; panel a), distance traveled to reach the platform (cm; panel b), swimming speed (cm.s^-1^; panel c), and the distance to platform (cm; panel d) are depicted. Performance of the four strains in the probe trial: the time spent in annuli (s; panel e) and quadrants (s; panel g), and the distance swum in annuli (cm; panel f) and quadrants (cm; panel h) are depicted. The means and standard errors of the means are shown. The lower left panel shows the quadrants, annuli, and the platform position during acquisition of the task.

The average escape latency did not differ by strain across the five successive daily training sessions (General mean: F_3,28 _= 1.02, NS). The time taken to find the platform decreased (Sessions: F_4,112 _= 95.40, p < 0.0001), but differently in the four strains (Sessions by Strain interaction: F_12,112 _= 3.07, p < 0.0009).

#### Acquisition, distance swum to escape onto platform (see Fig. 6b)

The strains differed in the average distance swum to reach the submerged platform (General mean: F_3,28 _= 7.26, p < 0.0009). All strains reduced the distance swum (Sessions: F_4,112 _= 71.59, p < 0.0001), but the decrease was different in the four stains (Sessions by Strain interaction: F_12,112 _= 2.43, p < 0.0077). Although the strains differed in the distance swum during sessions 1 to 4, they swam the same distance in session 5 [Fs_3,28 _and associated probabilities (p-values) for session 1 to 5, in that order: 3.58, 0.0262; 8.98, 0.0002; 6.12, 0.0025; 3.37, 0.0325; 0.22, NS].

Post-hoc comparisons between strains on the five successive daily sessions revealed that the BN rats performed better than the WKY rats in the first session. The performance of the F344 and Lewis rats was intermediate between that of the BN and WKY rats, from which they did not differ. In the second session, the BN rats had the best performance (shortest distance swum) and the F344 rats the worst (longest distance swum), with the WKY rats performing worse than the BN rats and the Lewis rats performing better than the F344 rats. The performance of the WKY and Lewis rats was not different. In the third session, the F344 rats performed worse than the BN and Lewis rats, which did not differ from WKY rats. In session 4, the BN rats performed better the F344 rats. The performance of the Lewis and F344 rats did not differ from one another or from that of the other two strains. In session 5, all strains had reached a similar performance level.

#### Acquisition, swimming speed (see Fig. 6c)

Both operational definitions of swimming speed revealed highly similar results, indicating that none of the strains spent a long time floating. The swimming speed, averaged across sessions, was different in the four strains (General mean: F_3,28 _= 8.23, p < 0.0004) and increased across sessions (Sessions: F_4,112 _= 74.62, p < 0.0001), again differently in the four strains (Sessions by Strain interaction: F_12,112 _= 4.06, p < 0.0001). Post-hoc comparisons between strains per session revealed that in the first session, the BN rats swam slower than the Lewis and F344 rats (the latter swam the fastest). The Lewis and WKY rats swam at the same speed. No strain differences in swimming speed were observed in sessions 2 and 3. In Session 4, the WKY and F344 rats swam faster than the Lewis and BN rats; the swimming speed of the WKY and F344 rats and of the Lewis and BN rats was not different. In session 5, the BN rats swam slower than the WKY and F344 rats, but not the Lewis rats.

Because of the differences in swimming speed, the escape latency is considered to be a biased measure of spatial learning in the MWM task. The distance swum to reach the platform, on the other hand, is taken as an unbiased measure of spatial learning.

#### Acquisition, distance to platform (see Fig. 6d)

The average distance to the platform was similar in the four strains (General mean: F_3,28 _= 2.59, NS). The mean distance to the platform decreased across successive sessions similarly in all strains (Sessions: F_4,112 _= 74.64, p < 0.0001; Sessions by Strain interaction: F_12,112 _= 1.54, NS).

#### Probe trial, time spent in annuli (see Fig. 6e)

There were no strain differences in the total time spent in the four annuli (F_3,28 _= 0.04, NS); however, all rats had a similar, strong bias for the training annulus (Annuli: F_3,84 _= 47.37, p < 0.0001; Annuli by Strain interaction: F_9,84 _= 0.66, NS).

#### Probe trial, distance swum in annuli (see Fig. 6f)

The four strains swam a similar total distance in the four annuli (F_3,28 _= 0.31, NS) but swam the furthest in the training annulus compared with the other three annuli (F_3,84 _= 54.21, p < 0.0001). The distance swum in the four annuli seemed to be different for the strains (Annuli by Strain interaction: F_9,84 _= 2.84, p < 0.0057) but a strain difference in the bias for the training annulus was not confirmed (F_3,28 _= 2.61, NS), although visual inspection of Fig. 6f suggests that the F344 rats swam a shorter distance in the training annulus than did the other three strains.

#### Probe trial, time spent in quadrants (see Fig. 6g)

All strains showed a clear and similar bias for the training quadrant (Quadrants: F_3,84 _= 57.99, p < 0.0001; Quadrants by Strain interaction: F_9,84 _= 1.61, NS).

#### Probe trial, distance swum in quadrants (see Fig. 6h)

All strains showed a clear bias for the training quadrant (Quadrants: F_3,84 _= 37.48, p < 0.0001; Quadrants by Strain interaction: F_9,84 _= 1.72, NS).

These data are consistent with the finding that the strains had reached a similar level of performance by the end of acquisition.

### Repeated acquisition in the Morris water tank

#### Platform escape latency (see Fig. 7a-d)

**Figure 7:**
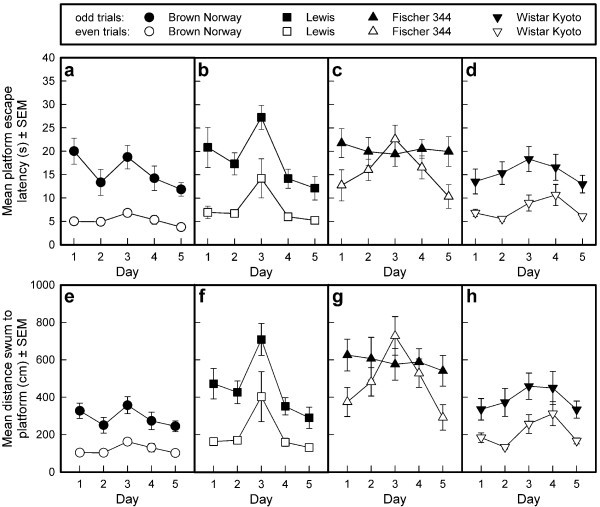
**Acquisition of the repeated acquisition task in the Morris water tank by four rat strains**. The mean platform escape latencies (s; panels a-d) and the distance traveled to reach the platform (cm; panels e-h) are depicted per strain as means and standard errors of the means (SEMs) of the odd and the even trials per session. The platform was situated in quadrant East, South, West, North, East in the five successive training sessions.

The average escape latency was different in the different strains (General mean: F_3,28 _= 11.39, p < 0.0001). Sidak post-hoc comparisons revealed that the average escape latency of the F344 rats was longer than that of the other three strains, which did not differ from one another. The change in mean escape latency across sessions was different in the four strains (Sessions: F_4,112 _= 7.69, p < 0.0001; Sessions by Strain interaction: F_12,112 _= 1.22, NS). Moreover, the escape latencies of the odd and the even trials were different (Trial pairs: F_1,28 _= 203.91, p < 0.0001) and influenced by strain (Trial pairs by Strains interaction: F_3,28 _= 5.64, p < 0.0037). The BN, Lewis, and WKY rats showed a consistent improvement from the odd to the even trials, indicating good working memory performance, whereas the F344 rats showed an inconsistent improvement. There was no interaction between Sessions and Trial pairs (F_4,112 _= 1.50, NS). However, there was a marginal interaction between Sessions, Trial pairs, and Strains (F_12,112 _= 1.65, 0.10 > p > 0.05), which probably reflects the inconsistent short-term memory performance of the F344 rats compared with that of the rats of the other three strains.

#### Distance swum to escape onto the platform (see Fig. 7e-h)

A similar picture as for the escape latencies emerged for the distance swum to escape onto the platform. Again, the F344 rats performed, on average, worse than the other three strains (General mean: F_1,28 _= 33.82, p < 0.0001), as confirmed by Sidak post-hoc comparisons. The BN rats performed better than the F344 and Lewis rats, but not better than the WKY rats. The average distance swum changed across sessions (Sessions: F_4,112 _= 8.18, p < 0.0001; Sessions by Strains interaction: F_12,112 _= 1.38, NS) and was longer in the odd trials than in the even trials (Trial pairs: F_1,28 _= 152.44, p < 0.0001). The size of this effect was different between strains (Trial pairs by Strains interaction: F_3,28 _= 3.94, p < 0.0182). While the BN, Lewis, and WKY rats showed a consistent improvement from the odd to the even trials, the F344 rats did not. There was no interaction between Sessions and Trial pairs (F_4,112 _= 1.23, NS). However, there was a marginal interaction between Sessions, Trial pairs, and Strains (F_12,112 _= 1.68, 0.10 > p > 0.05), which probably reflects the inconsistent short-term memory performance of the F344 strain compared with that of the other three strains.

#### Swimming speed (see Fig. 8a-d)

**Figure 8:**
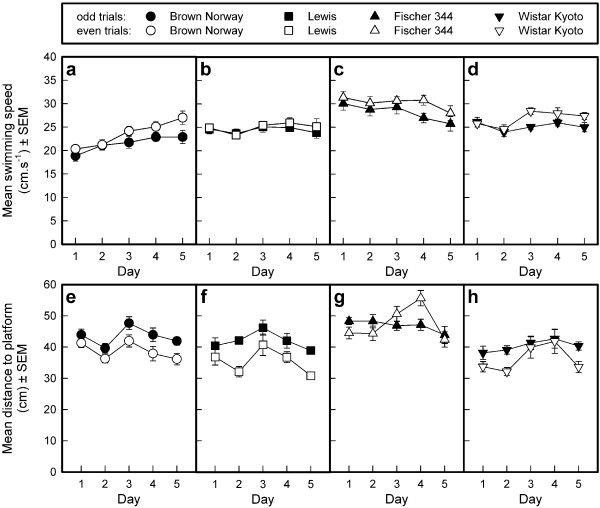
**Acquisition of the repeated acquisition task in the Morris water tank by four rat strains**. The mean swimming speed (cm.s^-1^; panels a-d) and the mean distance to the platform (cm; panels e-h) are depicted per strain as means and standard errors of the means (SEMs) of the odd and the even trials per session. The platform was situated in quadrant East, South, West, North, East in the five successive training sessions.

The two operational definitions of swimming speed revealed very similar results. The average swimming speed, calculated across all sessions, was different in the four strains (General mean: F_1,28 _= 12.11, p < 0.0001). Post-hoc Sidak comparisons confirmed that the F344 rats swam faster than the Lewis and BN rats, but not faster than the WKY rats. The BN rats swam the slowest, differing from the F344 and WKY rats but not the Lewis rats. The average swimming speed changed across sessions (Sessions: F_4,112 _= 4.44, p < 0.0023;) and to a different extent in the four strains (Sessions by Strains interaction: F_12,112 _= 4.73, p < 0.0001), probably because the BN rats swam faster across sessions whereas the swimming speed of the other strains remained the same or decreased slightly across sessions. The swimming speed was, on average, faster during odd than during even trials (Trial pairs: F_1,28 _= 28.91, p < 0.0001; Trial pairs by Strains interaction: F_3,28 _= 1.54, N). This difference varied across sessions (Trial pairs by Sessions interaction,: F_4,112 _= 5.47, p < 0.0005) but was not different in the different strains (Sessions by Stains by Trial pairs interaction: F_12,112 _= 1.18, NS).

#### Mean distance to platform (see Fig. 8e-h)

Averaged across all sessions, the distance to the platform was different in the four strains (General mean: F_1,28 _= 13.36, p < 0.0001). The F344 rats swam at the greatest distance from the platform (note that the analysis was performed with respect to the changing position of the escape platform in the five sessions) whereas the other three strains did not differ from each other, as confirmed by Sidak post-hoc comparisons. The mean distance to the platform changed between sessions (Sessions: F_4,112 _= 10.56, p < 0.0001), similarly in the four strains (Sessions by Strains interaction: F_12,112 _= 1.25, NS), being the greatest during odd trials (Trial pairs: F_1,28 _= 46.78, p < 0.0001), but this effect was strain dependent (Trialpairs by Strains interaction: F_3,28 _= 7.99, p < 0.0005). The F344 rats did not consistently swim at a shorter distance from the platform during odd than during even trials. The difference between odd and even trials fluctuated between sessions (Trial pairs by Sessions interaction: F_4,112 _= 4.03, p < 0.0043). This fluctuation was different in the different strains (Sessions by Stains by Trial pairs interaction: F_12,112 _= 1.93, p < 0.0376), probably because the difference between the average distance to the platform in odd and even trials reversed in the F344 rats in the third and fourth sessions.

### Passive avoidance task

One of the F344 rats did not enter the dark compartment during the shock session, and consequently did not receive a shock. Therefore, the data for this animal were not included in the analyses.

#### Step-through latency to enter the dark compartment (see Fig. 9)

**Figure 9:**
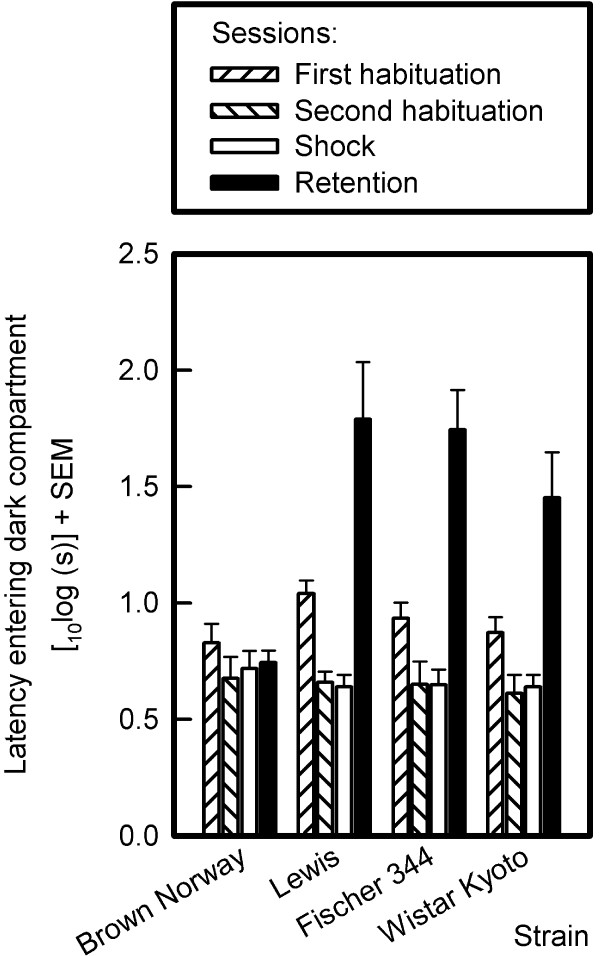
**Entry latencies of BN, Lewis, F344 and WKY rats in the first and second habituation session, the shock session, and the retention session in the passive avoidance task**. The logarithmically transformed mean latencies and standard errors of the means (SEMs) are depicted. Means represent 8 animals per strain, except for the F344 strain, where the number of animals in analysis was 7, because one F344 rat did not enter the dark compartment during the shock session.

The step-through latencies did not differ in the four strains during the habituation and shock sessions (first habituation session: F_3,27 _= 1.88, NS; second habituation session: F_3,27 _= 0.13, NS; shock session: F_3,27 _= 0.38, NS) but did in the retention session (F_3,27 _= 7.25, p < 0.001). Post-hoc Sidak pairwise comparisons revealed that the BN rats had shorter step-through latencies than the Lewis and F344 rats but not the WKY rats. The BN rats would thus appear not to have remembered the aversive shock.

### 5-HT, 5-HIAA, and 5-HT turnover in hippocampus and cortex

#### Hippocampus

**Figure 10:**
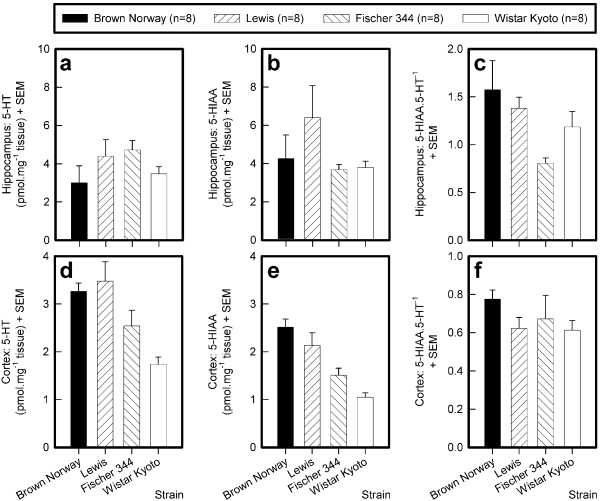
**Hippocampal and cortical levels of 5-HT and 5-HIAA, and serotonin turnover (5-HIAA.5-HT^-1^) in four rat strains**. The means and the standard errors of the means (SEMs) are depicted.

The hippocampal 5-HT content (F_3,28 _= 1.33, NS; see Fig. 10a) and 5-HIAA content (F_3,28 _= 1.45, NS; see Fig. 10b) were not different in the different strains but 5-HT turnover was (F_3,28 _= 3.23, p < 0.0373; see Fig. 10c). Hippocampal 5-HT turnover was higher in the BN than in the F344 rats. 5-HT turnover in the Lewis and the WKY rats was intermediate and not different between the two strains, nor was it different from that of the other two strains, as confirmed by Sidak post-hoc comparisons

#### Cortex

The cortical 5-HT content (F_3,28 _= 7.63, p < 0.0007; see Fig. 10d) and the 5-HIAA content (F_3,28 _= 13.31, p < 0.0001; see Fig. 10e) were different in the different strains. Post-hoc comparisons revealed that the 5-HT content was lowest in the WKY strain and highest in the Lewis and BN strains. The F344 rats had intermediate levels: their 5-HT content did not differ from that of the other three strains. The 5-HIAA content was highest in the BN rats. This strain differed from the F344 and WKY strains, which did not differ from one another. 5-HT turnover in the cortex was not different between strains (F_3,28 _= 0.96, NS; see Fig. 10f).

### Correlation analysis and principal component analysis

The Spearman and Pearson correlation coefficients and their associated probabilities are summarized in Table 1. Most correlations were weak (r < 0.50); very few were moderate (0.50 > r > 0.80), and even less were high (p ≥ 0.80). Below we describe some of the relationships.

**Table 1:** Correlations between measures of emotional reactivity, performance in learning and memory tasks, and serotonergic measures.

			**Emotional reactivity (ER)**									**Learning and memory (L&M)**					**Serotonergic (5-HT) measures**		
			**1**	**2**	**3**	**4**	**5**	**6**	**7**	**8**	**9**	**10**	**11**	**12**	**13**	**14**	**15**	**16**	**17**
					
ER	1) OF time in corners (mean 5 days)	r	1.00	-0.06	-0.21	-0.21	-0.27	0.14	0.35	-0.09	-0.19	0.07	-0.13	0.03	0.12	0.15	0.27	0.05	-0.06
		p		0.73	0.24	0.24	0.13	0.44	0.05	0.61	0.29	0.70	0.47	0.89	0.51	0.42	0.13	0.80	0.76
	2) OF line crossings (mean 5 days)	r	-0.04	1.00	**0.73**	**-0.40**	-0.17	**0.40**	0.20	**0.43**	**0.48**	**-0.55**	0.14	**0.37**	**0.60**	-0.12	**0.54**	0.07	0.21
		p	0.82		**<0.01**	**0.02**	0.36	**0.02**	0.28	**0.01**	**0.01**	**0.00**	0.45	**0.04**	**0.00**	0.52	**0.00**	0.70	0.24
	3) OF rearings & leanings (mean 5 days)	r	-0.17	**0.82**	1.00	**-0.49**	-0.18	**0.66**	-0.12	**0.39**	**0.72**	**-0.69**	0.20	**0.46**	**0.55**	**-0.48**	**0.43**	0.24	**0.44**
		p	0.36	**<0.01**		**0.00**	0.31	**<0.01**	0.52	**0.03**	**<0.01**	**<0.01**	0.27	**0.01**	**0.00**	**0.01**	**0.01**	0.19	**0.01**
	4) EPM percent time in open arms (day 1)	r	-0.31	**-0.48**	**-0.48**	1.00	**0.83**	-0.33	0.04	-0.16	**-0.39**	**0.49**	-0.23	-0.23	**-0.54**	**0.57**	**-0.46**	-0.19	-0.32
		p	0.08	**0.01**	**0.01**		**<0.01**	0.07	0.84	0.38	**0.03**	**0.00**	0.20	0.21	**0.00**	**0.00**	**0.01**	0.30	0.08
	4) EPM percent entries open arms (day 1)	r	-0.30	-0.24	-0.25	**0.86**	1.00	-0.20	0.02	-0.21	-0.16	0.32	-0.24	-0.03	**-0.44**	**0.41**	**-0.38**	-0.25	-0.30
		p	0.09	0.19	0.16	**<0.01**		0.28	0.92	0.24	0.39	0.07	0.19	0.89	**0.01**	**0.02**	**0.03**	0.16	0.10
	6) EPM rearings & leanings (day 1)	r	0.13	**0.51**	**0.63**	**-0.37**	-0.22	1.00	0.04	0.18	**0.35**	**-0.44**	0.08	**0.41**	0.25	-0.17	**0.45**	0.21	0.25
		p	0.47	**0.00**	**0.00**	**0.04**	0.24		0.81	0.32	**0.05**	**0.01**	0.65	**0.02**	0.17	0.37	**0.01**	0.25	0.17
	7) cLDB time in dark (mean 3 days)	r	0.30	0.21	-0.05	0.00	0.01	0.02	1.00	-0.30	**-0.50**	0.08	-0.22	0.00	0.00	**0.37**	0.12	-0.26	**-0.38**
		p	0.09	0.24	0.80	0.98	0.95	0.90		0.10	**0.00**	0.65	0.22	0.99	0.98	**0.04**	0.50	0.15	**0.03**
	8) cLDB line crossings (mean 3 days)	r	-0.07	0.13	0.09	-0.09	-0.15	0.00	-0.24	1.00	**0.58**	**-0.40**	0.23	0.11	**0.34**	-0.17	0.20	0.16	**0.39**
		p	0.70	0.49	0.61	0.62	0.40	1.00	0.20		**0.00**	**0.02**	0.21	0.55	**0.05**	0.36	0.26	0.38	**0.03**
	9) cLDB rearings & leanings (mean of 3 days)	r	-0.20	**0.52**	**0.69**	**-0.37**	-0.30	**0.42**	**-0.43**	**0.52**	1.00	**-0.67**	0.21	**0.45**	**0.45**	**-0.47**	0.30	0.34	**0.43**
		p	0.27	**0.00**	**<0.01**	**0.04**	0.10	**0.02**	**0.01**	**0.00**		**<0.01**	0.25	**0.01**	**0.01**	**0.01**	0.10	0.06	**0.01**
																			
L&M	10) sMWM distance swum (mean 5 days)	r	0.14	**-0.63**	**-0.61**	**0.42**	0.28	**-0.42**	0.12	-0.27	**-0.70**	1.00	-0.27	**-0.55**	**-0.47**	**0.37**	**-0.38**	**-0.45**	**-0.54**
		p	0.46	**0.00**	**0.00**	**0.02**	0.12	**0.02**	0.50	0.14	**<0.01**		0.13	**0.00**	**0.01**	**0.04**	**0.03**	**0.01**	**0.00**
	11) sMWM distance swum (lin. trend across 5 days)	r	0.02	0.02	0.11	-0.24	-0.28	0.09	-0.13	0.09	0.13	-0.05	1.00	-0.21	0.25	-0.34	0.20	0.14	0.26
		p	0.93	0.91	0.54	0.19	0.12	0.61	0.49	0.63	0.47	0.80		0.24	0.17	0.06	0.26	0.43	0.16
	12) sMWM probe trial time in training annulus	r	0.10	0.24	0.28	-0.24	-0.11	0.28	-0.25	-0.02	**0.39**	**-0.39**	-0.33	1.00	0.05	0.02	0.19	0.23	0.14
		p	0.60	0.19	0.12	0.20	0.55	0.12	0.16	0.91	**0.03**	**0.03**	0.06		0.80	0.92	0.31	0.21	0.44
	13) raMWM delta (odd-even trials) (mean 5 days)	r	0.13	**0.56**	**0.52**	**-0.51**	**-0.37**	0.21	-0.11	0.14	**0.42**	**-0.37**	0.20	-0.06	1.00	**-0.38**	**0.42**	0.23	**0.42**
		p	0.48	**0.00**	**0.00**	**0.00**	**0.04**	0.24	0.54	0.45	**0.02**	**0.04**	0.27	0.74		**0.03**	**0.02**	0.20	**0.02**
	14) PA log latency entry dark, retention	r	0.13	-0.15	**-0.43**	**0.41**	**0.36**	-0.13	0.31	-0.12	**-0.40**	0.24	-0.35	0.13	-0.30	1.00	**-0.36**	-0.07	**-0.37**
		p	0.50	0.42	**0.01**	**0.02**	**0.04**	0.49	0.09	0.53	**0.03**	0.19	0.06	0.50	0.10		**0.05**	0.70	**0.04**
																			
5-HT	15) 5-HT turnover (5-HIAA.5-HT^-1^) in hipp.	r	0.18	**0.46**	**0.38**	-0.33	-0.17	**0.36**	0.01	0.01	0.22	**-0.35**	0.23	0.16	0.12	-0.30	1.00	0.33	0.30
		p	0.31	**0.01**	**0.03**	0.06	0.35	**0.05**	0.95	0.96	0.23	**0.05**	0.21	0.39	0.52	0.10		0.07	0.10
	16) 5-HT in cortex	r	0.07	0.14	0.25	-0.16	-0.17	0.25	-0.29	0.14	0.32	**-0.42**	0.06	0.28	0.26	0.06	0.27	1.00	**0.76**
		p	0.70	0.46	0.17	0.39	0.34	0.18	0.10	0.44	0.07	**0.02**	0.73	0.12	0.16	0.74	0.14		**<0.01**
	17) 5-HIAA in cortex	r	-0.04	0.31	**0.42**	-0.26	-0.26	0.26	**-0.43**	0.22	**0.42**	**-0.52**	0.16	0.15	**0.51**	-0.20	0.22	**0.78**	1.00
		p	0.82	0.08	**0.02**	0.15	0.16	0.16	**0.02**	0.22	**0.02**	**0.00**	0.38	0.40	**0.00**	0.28	0.23	**<0.01**	

The correlation coefficients (r) (Spearman: above diagonal; Pearson: below diagonal) and the associated probabilities (p) are shown. Note: all correlations are based on the data of 32 animals except the correlations with 'PA log latency entry dark, retention', which are based on the data of 31 animals. Correlation coefficients with associated probabilities < 0.05 are printed bold.

#### Correlations between the three tasks measuring emotional reactivity

The mean time spent in the safest area of the OF (corners) and the cLDB (dark compartment) was unrelated to horizontal activity (line crossings) but was positively correlated with vertical activity (rearing and leaning; for the Spearman correlation 0.1 > p > 0.05). Horizontal activity in the OF and cLDB was positively correlated with rearing and leaning. In the EPM, percent time spent in the open arms was positively correlated with percent entries in open arms on day 1. Horizontal activity and vertical activity were correlated in the OF and cLDB but not in the EPM.

#### Correlations between the aversively motivated learning and memory tasks

Surprisingly, the average performance (reflected by the average distance swum to reach the platform) in the sMWM task was not associated with the speed of learning (reflected by the linear trend component of the distance swum to reach the platform). However, the average sMWM performance was weakly correlated with bias for the training annulus, and with raMWM performance: the shorter the distance swum to reach the platform during acquisition, the more time a rat spent in the training annulus during the probe trial and the larger the difference between the odd and even trials in the raMWM task. Retention performance in the PA was weakly correlated with distance swum in the sMWM task and with the difference score between odd and even trials in the raMWM task. The better PA retention, the longer the distance the rats swam to reach the platform, and the smaller the difference between odd and even trials in the raMWM task.

#### Correlations between activity, emotional reactivity, and cognitive performance

Rearing in each of the three tests of emotional reactivity was correlated with several measures in the learning and memory tasks. In addition, horizontal activity in the OF was related with performance in the three learning and memory tasks. Rearing in the three tests of emotional reactivity was negatively correlated with the mean distance swum in the sMWM task: rats that were more active in these tests swam a shorter distance in the sMWM to escape onto the submerged platform. None of the measures in the OF, cLDB, and EPM was correlated with the linear trend component of the distance swum in the sMWM, which can be considered to reflect the speed of learning the task. Thus the speed of learning would appear to be independent of both activity and emotional reactivity.

The difference in performance (delta) in the odd and even trials in the raMWM was weakly correlated with rearing in the OF and the cLDB, indicating that rats that showed the greatest improvement from the first to the second trial (an index for spatial working memory) also showed more vertical activity. The percent time spent in the open arms of the EPM was negatively correlated with working memory performance in the raMWM: rats that spent less time in the open arms had a better working memory performance. The retention performance in the PA task was negatively correlated with rearing in the OF and the cLDB, i.e. less active rats had a better PA retention.

#### Correlations between the serotonergic measures

5-HT and 5-HIAA levels in the cortex were positively correlated.

#### Correlations between the serotonergic measures and behavior in the tests for emotional reactivity

A higher 5-HT turnover in the hippocampus was positively correlated with vertical activity in OF and EPM, and with horizontal activity in the OF.

#### Correlations between the serotonergic measures and performance in the learning and memory tasks

A higher 5-HT turnover in the hippocampus, a higher level of 5-HIAA in the hippocampus, and a higher level of 5-HT in the cortex appeared to be associated with better performance in the sMWM, as indicated by the shorter mean distance swum to reach the platform. The 5-HIAA level in the cortex was correlated with a better working memory performance in the raMWM.

#### Principal component analysis

Results of principal component analyses of 17 measures as observed and of corresponding residuals after correction for strain effects are summarized in Table 2. Both PCAs yielded five factors, cumulatively explaining 73% and 70% of the total variation. Different measures generally separated out well on different factors. Factor 1 produced by PCA of the measures had high negative loadings for rearings in the cLDB, OF, and EPM, for line crossings in the OF, and for raMWM delta, and the hippocampal 5-HT turnover. The mean distance swum in the sMWM loaded positively on factor 1. Serotonergic measures in the cortex loaded exclusively on factor 2, together with the average time spent in the dark compartment in the cLDB. Factor 3 was related to the linear trend in the distance swum, the time spent in the training annulus in the sMWM probe trial, and the latency to enter the dark compartment in the PA task. The fourth factor summarized the behavioral measures recorded in the cLDB, together with the time spent in the corners in the OF. Factor 5 was dominated by the time spent in the corners in the OF and the percent time spent in and entries of the open arms in the EPM.

**Table 2:** Results of the principal component analysis.

**Test/essay measure**	**A) Loadings on the first five factors extracted by PCA, varimax rotation**					**B) Loadings on the first five factors extracted by PCA, varimax rotation, after correction for strain effects: residuals**				
		
	**Factor 1**	**Factor 2**	**Factor 3**	**Factor 4**	**Factor 5**	**Factor 1**	**Factor 2**	**Factor 3**	**Factor 4**	**Factor 5**
		
**open field**										
Time in corners (mean 5 days)				0.73	-0.50				-0.78	
Line crossings (mean 5 days)	-0.92					-0.87				
Rearings (mean 5 days)	-0.91					-0.90				
**elevated plus maze**										
Percent time open arms (day 1)					0.86				0.83	
Percent entries open arms (day 1)					0.89				0.87	
Rearings (day 1)	-0.69					-0.64				
**circular Light-dark box**										
Time in dark (mean 3 days)		-0.56		0.59				0.68		
Line crossings (mean 3 days)				-0.62				-0.71		
Rearings (mean 3 days)	-0.50			-0.67		-0.63		-0.67		
**standard MWM**										
Distance swum (mean 5 days)	0.64					0.72				
Distance swum (linear trend)			0.79				0.62			
Probe trial time training annulus			-0.74							-0.55
**repeated acquisition MWM**										
Delta (odd minus even trials)	-0.51									0.75
**passive avoidance**										
Log latency entry dark, retention			-0.57				-0.60			
**serotonin**										
5-HT turnover in hippocampus	-0.51									
5-HT cortex		0.90					-0.81			
5-HIAA cortex		0.85					-0.75			
		
**Variance explained (%)**	32.0	12.3	11.5	9.0	8.0	24.4	15.1	12.4	10.0	8.1

Loadings, higher than 0.5, on the first five factors extracted by principal component analysis (PCA), after varimax rotation (A), and of residuals obtained after correction for the effect of strain (B), of measures of emotional reactivity, spatial learning and memory in the standard and the repeated acquisition versions of the MWM task, the passive avoidance task, and serotonergic measures in hippocampus and cortex of 32 rats. The percent of total variation explained by each factor is also shown.

The loading patterns of three of the five factors were largely maintained after correction for strain. With the exception of raMWM and the hippocampal 5-HT turnover, factor 1 had high and same-sign loadings for the same measures after correction for strain. Thus, in both cases, rats with low scores on factor 1 reared a lot in the cLDB, OF and EPM, made many line crossings in the OF, and swam a relatively short distance before finding the submerged platform in the sMWM. The loading pattern of factor 4 extracted in the PCA of the measures as observed was similar to the loading pattern of factor 3, extracted in the PCA of residuals. The loading pattern of factor 5 obtained with PCA of the observed data was identical to the loading pattern of factor 4 obtained with PCA of residuals. High scores on either factor 5 without or factor 4 with correction for strain effects were associated with high proportion of time spent in and entries made into the open arms in the EPM, and with a relatively short time spent in the corners of the OF, and vice versa.

## Discussion

The four strains of rats performed differently in the behavioral tests, with the F344 rats having the poorest performance in both versions of the Morris water escape task whereas the BN rats had a very good performance in the Morris water escape task but a very poor performance in the passive avoidance task. None of the rats had sensory or motor deficits that could cause behavioral or motor problems. Correlation analysis and principal component analysis did not provide convincing support that the OF, EPM, and cLDB measure the *same *underlying trait. Moreover, we did not find evidence that emotional reactivity modulates cognitive performance in aversively motivated tasks.

### Emotional reactivity

Following the recommendation that emotional reactivity should be evaluated in a battery of tests, not just a single test [81,82], we tested the four rat strains in the OF, the EPM, and the cLDB.

#### Open field

The many different designs of the OF with respect to its shape (square [83] vs. circular [15,84]), presence or absence of sidewalls ("elevated open field"; [85]), and size [86], make it difficult to compare the results of various studies and may, at least partly, be responsible for the inconsistent results reported [87]. Recently, however, Eilam [86] demonstrated that OF behaviors are fairly robust across a large range of OF sizes. The time spent in the center may be less sensitive than the time spent in the corners as index of emotional reactivity or anxiety, because the time spent in the center decreased to near zero in most rat strains across testing days, whereas the time spent in the corners increased and the difference between strains increased with repeated testing (see also Fig. 3a). Although the four corner squares cover only 11% of the floor surface of the OF, the rats spent between 30% and 75% of the time in the corners (percent of total session duration, for days 1 to 5, in that order: BN, 35.7, 42.8, 45.3, 57.7, 57.8; Lewis, 33.5, 52.5, 66.1, 75.0, 71.5; F344, 30.1, 49.9, 55.5, 50.0, 46.8; and WKY, 34.4, 55.9, 56.2, 78.3, 70.0), corroborating earlier findings [12,21]. If animals spend only a very short time in the most aversive area of the test environment (the center of the OF) [45], it is only possible to detect the anxiolytic effects of experimental treatments, whereas both anxiogenic and anxiolytic effects of treatments can be detected by measuring time rats spend in the 'safest' part of the OF (the corners). Our findings suggest that the sensitivity of this measure to detect changes in emotional reactivity will not be restricted by floor of ceiling effects.

The BN rats showed a relatively low avoidance of the center of the OF, corroborating the findings of Ramos and colleagues [88], although our findings for Lewis and F344 rats do not corroborate their findings for the same strains. This may reflect a difference between substrains obtained from different commercial breeders (Ramos and coworkers: Iffa Credo [88]; this study: Charles River).

#### Elevated plus maze

An increased occupancy of the enclosed arms and a decreased entry into the open arms are considered to indicate higher emotional reactivity, whereas the number of closed arm entries is considered an index of activity that is independent of anxiety [24]. In contrast, we found that the time spent in the open arms was positively correlated with the number of open arm entries - the rats that spent more time in the open arms also made more open arm entries. Our data are in agreement with those of Shepard and Myers [89], who compared the EPM behavior of F344 and WKY rats.

On the basis of the first EPM test, BN rats could be considered the most anxious and the F344 rats the least anxious strains, with Lewis and WKY rats having an intermediate position; however, these strain differences were no longer seen on the second day of testing, because the F344 rats spent considerably less time in the open arms and made considerably fewer open arm entries. Pellow and co-workers have suggested that behavior in the EPM is independent of novelty because it is stable across successive sessions [23]. Our findings support this notion for all strains except the F344 strain. If the marked difference in the behavior of the F344 rats from session 1 to session 2 is taken to reflect an acquired avoidance response (i.e., learning to avoid entering potentially dangerous part of the apparatus [68]), then the ability of F344 rats to learn is superior to that of the other strains tested. However, the difference in behavior can also be ascribed to anxiety sensitization of a phobic state [90].

Although Lewis rats have been reported to be very anxious in the EPM (e.g., [53]), we did not find this to be the case. Instead, we found the Lewis and WKY rats to have a similar performance, as also reported by Ramos et al [53]. Most strain differences, with the exception of less rearing activity in F344 rats, were no longer detected on the second day of testing, corroborating the notion that pre-exposure to the EPM affects subsequent testing in this test [25,91]. F344 rats showed the strongest adaptation to the EPM, whereas the other three strains showed virtually no change from the first to the second day of testing.

#### Circular light dark box

We designed a circular Light-Dark preference Box (cLDB) [12] that, apart from differences in light intensity, lacks any spatial cues for the light compartment and the dark compartment and has a sharp transition from the light to the dark compartments. Many light-dark boxes provide spatial cues for the transition from the light to the dark (e.g. a small door in the wall separation the light and dark compartment, an open, large light compartment vs. a small, closed dark compartment; [92,93]). We believe that the lack of these cues makes the cLDB a better instrument to measure light avoidance or dark preference than the light-dark boxes normally used in rodent studies (see also [94]). The equal size of both compartments also makes it possible to compare locomotor activity in both compartments directly.

We found that WKY and Lewis rats spent more time in the dark compartment than the F344 and BN rats did, whereas there were no strain differences in horizontal activity. Testing in the cLDB thus may have affected the emotionality of the latter two strains less than that of the WKY and the Lewis strains. Our results do not corroborate the strain differences in reactivity found in the other two tests of emotional reactivity. Avoidance of exposed areas (the center of the OF and the open arms of the EPM) thus may be controlled differently from avoidance of brightly lit areas (the light compartment of the cLDB). This finding contradicts the notion that the OF, EPM, and cLDB measure the same underlying trait(s).

#### Defection as index of emotional reactivity

If defecation is taken to reflect emotionality [15,16], then BN rats appeared to have the highest emotional reactivity, followed by F344 rats. Lewis and WKY rats were the less reactive strains. The OF may induce more anxiety than the other two tests if defecation is considered a valid index of emotionality in the OF test [95]; however, others have questioned the validity of OF defecation as index of emotionality of rats [96,97].

#### Putative effects of testing order

We tested the rats in successive sessions and it is possible that learning processes affected the animal's behavior in subsequent sessions [1,24] or in subsequent tests [13,98]. Integrated tests have been developed to minimize potential learning effects (e.g., the modified holeboard task [2]; the concentric square field [98]; and an integration of the OF, EPM, and LDB [77]). However, Ramos et al. found similar results when comparing an integrated OF-EPM-LDB test with a series of successive OF, EPM, and LDB tests [77]. One caveat of the three tests used to assess emotional reactivity is that they are sensitive to the environment and the test conditions applied [13,99]. We agree with a notion of Bouwknecht and Paylor [45] that strains can be (re)tested repeatedly if testing is performed in the same order and if the impact of previous testing is low or absent.

### Learning and memory

#### Standard Morris water escape task

The BN rats acquired the standard Morris water escape task very quickly, corroborating their good spatial learning abilities found in other studies (Morris task [12,62] and hole board task [63]), whereas the F344 rats were slightly slow to acquire the task, as reported earlier [11], but by the end of testing they had attained a similar performance level as the other three strains. The BN and F344 strain differences were smaller than reported previously [12]. Unlike other studies, we did not observe the WKY rats to float in the MWM tasks [39,100].

#### Repeated acquisition task in the Morris maze

The BN, Lewis, and WKY rats performed the short-term memory task well, corroborating findings for WKY rats [101] although Clement and colleagues [100] found no improvement across trials in a similar task using WKY rats. The performance of F344 rats was unstable across sessions, and they did not show improvement from odd to even trials in the second, third, and fourth sessions. Thus the spatial working memory of F344 rats is already poor by 6 months of age, as reported previously by Shukitt-Hale, Mouzakis and Joseph [102].

#### Passive avoidance

The BN rats did not show retention of the passive avoidance task, i.e. they entered the dark compartment as rapidly as during the habituation sessions and the shock session. This corroborates earlier findings that the BN strain performs shock-motivated tasks poorly [12,103]. In an earlier study, we did not observe a flinch reaction in BN rats, even at a shock intensity as high as 0.5 mA [12]. Moreover, BN rat have been found to show only a weak reaction to stressors (see above). Surprisingly, strain differences were relatively small in the three learning and memory tasks. The F344 strain showed the poorest performance in both versions of the MWM task, whereas BN rats performed very poorly in the passive avoidance task, but very well in the MWM tasks.

### Serotonergic measures

Previously, we have shown that 5-HT1A receptor agonists, which lower 5-HT turnover in postsynaptic brain areas (dorsal hippocampus and cortical areas, etc.) via stimulation of the raphe 5-HT1A autoreceptors, produce anxiolytic effects in the EPM, defensive burying test, and conditioned freezing test [104-106]. The lower hippocampal 5-HT turnover in F344 rats as compared to BN rats was consistent with the finding that F344 rats exhibited less anxiety (i.e., they showed more open arm exploration) than BN rats. Lieben and colleagues found that acute depletion of tryptophan, the precursor of 5-HT, did not affect the performance of rats in the sMWM and in tests measuring affective behavior [107]. We found that the level of 5-HIAA and 5-HT in the cortex and 5-HT turnover in the dorsal hippocampus were correlated negatively with the performance of the rats in the sMWM. This is probably because increased stimulation of postsynaptic 5-HT1A receptors by 5-HT in the dorsal hippocampus and cortical areas results in hyperpolarization of these target neurons, *making *them *less *likely to fire an action potential [46]. Moreover, the binding of 5-HT to postsynaptic 5-HT1A receptors also inhibits cholinergic neurotransmission in magnocellular nucleus basalis neurons projecting to the somatosensory cortex [108].

### Correlation analysis and principal component analysis

The lack of (negative) correlations between time spent in the most protected areas of the OF and cLDB and horizontal activity suggest that these variables are independent, and that the time spent in the most protected areas of the apparatus may be considered a measure of emotional reactivity. This contrasts with the notion that emotionally reactive animals spend more time in the most protected areas of the apparatus *and *show decreased locomotor activity [14,20,21]. We found a moderate, positive correlation between the time spent in the open arms and the number of open arm entries in the EPM, in agreement with the finding of Fernandes and File [25] that both variables load positively on the same 'anxiety' factor. This relationship was found in EPMs with and without ledges around the open arms.

Rats that performed better in the sMWM, i.e. swam a shorter distance to escape onto the submerged platform, also showed a stronger bias for the training annulus in the probe trial. This correlation was weak and corroborates earlier findings [109]. Also, the rats that performed better in the sMWM showed a better short-term memory performance in the raMWM. We found that a higher level of emotional reactivity facilitated retention of the passive avoidance task, corroborating findings by Ribeiro and co-workers [110].

Analysis of the correlation between measures believed to indicate emotional reactivity and measures of learning and memory did not support the notion that emotional reactivity modulates the cognitive processes that are involved in the MWM and PA tasks nor the assumption that emotional reactivity is a confounding variable for learning and memory [10-12].

Principal component analysis provided a concise summary of the underlying correlation matrix and clearly revealed a multifactorial picture. This is consistent with the findings of several other studies involving laboratory rodents tested in different paradigms (see [77,87]). We identified two dimensions that consistently summarized measures recorded in different paradigms. One dimension was dominated mainly by rearing in the cLDB, OF, and EPM, line crossings in the OF, and the distance swum in the sMWM. The other dimension was associated with the time spent in the corners in the OF and the time spent in the open arms and number of open arm entries in the EPM. The loading patterns of these two dimensions are similar to those reported elsewhere, and are generally believed to reflect locomotor activity and emotional reactivity, respectively [77,78]. Interestingly, while we found the emotional factor to be associated with OF and EPM measures, Ramos and co-workers [77] found locomotion in the OF and the time spent in and entries of the open arms in EPM to load on different factors. The loading pattern of the various behavioral measures was largely maintained after correction for the effect of strain (Table 2A, Factors 1 and 5, compared with 2B, Factors 1 and 4, respectively). This indicates that covariation of these measures was similar within and between rat strains, thus providing support for the assumption that these measures might be genetically linked.

Behavioral measures recorded in the cLDB and the OF showed different associations before and after correction for the effect of strain. PCA on the observed data resulted in a factor with high loadings on the time spent in the dark, rearings, and line crossings in the cLDB, and the time spent in the corner in the OF (Table 2A, Factor 4). After correction for strain, the time spent in the corners of the OF was no longer related to the behaviors observed in the cLDB (Table 2B, Factor 3). Similarly, serotonerigc measures were not consistently associated with behavioral measures before and after correction for the effect of strain. This suggests that the relationships between serotonergic and behavioral measures are not necessarily genetically linked in animals of the same strain, and that biological differences between various rat strains may result from a fortuitous selection of unrelated characteristics. Systematic breeding experiments, involving the creation of nonsegregating as well as segregating crossings (F2 and backcrosses), are necessary to identify genetic links between behavioral and neurochemical measures of emotional reactivity [87,111].

### Validity of the set of three tests of emotional reactivity

We attempted to corroborate the construct validity of the three tests by using rat strains that were expected to differ in emotional reactivity. The construct validity was examined by calculating the correlations across the four rat strains, supplemented with the PCA, to determine the convergence of measures that are presumed to tap the same construct, namely, emotional reactivity assessed in the OF, EPM and cLDB tests. Indices of emotional reactivity in the OF, EPM and cLDB, such as an increased time spent in the 'safest' area of the apparatus, or a decrease in the time spent in the aversive area(s) of the apparatus, should be strongly correlated if they all measure the same trait [21]. Neither the correlation analysis nor the principal component analysis provided convincing support for the notion that the three tests measure the *same *underlying trait in the rat strains tested.

## Conclusions

Animal experiments are often performed to understand neurobiological mechanisms of cognition and emotion, on the assumption that similar mechanisms are active in humans. Here we show that the behavioral outcome of an animal experiment is dependent on the test and animal strain used. Thus experiments should be performed with a battery of tests and with different animal strains to increase the generalizability and the translational value of findings i.e. whether the behavioral outcome is a general character that is independent of the specific strain(s) and/or test(s) used [112]. The strain differences found in the present study are generally in line with earlier findings [12] and underline the complexity of strain differences measured in different tests and tasks. Concepts such as 'emotional reactivity' and 'learning and memory' cannot be tapped adequately with only one behavioral test and our results confirm the need for multiple testing [113]. This study does not support our hypothesis that the level of emotional reactivity modulates cognitive performance in aversively motivated tasks.

## Competing interests

The authors declare that they have no competing interests.

## Authors' contributions

FJS and TS conceived the study. CGR performed and described the results of the principal components analysis, and SMK coordinated, analyzed and reported the serotonergic measurements. FJS drafted the manuscript, and all authors participated in writing the final version of the manuscript.
